# Exploring health equity integration among health service and delivery systems in Nova Scotia: perspectives of health system partners

**DOI:** 10.1186/s12939-024-02256-7

**Published:** 2024-08-26

**Authors:** Joshua Yusuf, Ninoshka J. D’Souza, Hilary A.T. Caldwell, Sarah Meaghan Sim, Mark Embrett, Sara F.L. Kirk

**Affiliations:** 1 School of Health and Human Performance, Dalhousie University, 6230 South St, Halifax, NS B3H 4R2 Canada; 2 Healthy Populations Institute, Dalhousie University, 1318 Robie St, Halifax, Halifax, NS B3H 3E2 Canada; 3 Nova Scotia Health, Halifax, Canada

**Keywords:** Health equity, Delivery of Health Care, Attitude of Health personnel, Health priorities

## Abstract

**Background:**

Achieving health equity is important to improve population health; however, health equity is not typically well defined, integrated, or measured within health service and delivery systems. To improve population health, it is necessary to understand barriers and facilitators to health equity integration within health service and delivery systems. This study aimed to explore health equity integration among health systems workers and identify key barriers and facilitators to implementing health equity strategies within the health service and delivery system in Nova Scotia, ahead of the release of a Health Equity Framework, focused on addressing inequities within publicly funded institutions.

**Methods:**

Purposive sampling was used to recruit individuals working on health equity initiatives including those in high-level leadership positions within the Nova Scotia health system. Individual interviews and a joint interview session were conducted. Topics of discussion included current integration of health equity through existing strategies and perceptions within participant roles. The Consolidated Framework for Implementation Research (CFIR) was used to guide coding and analysis, with interviews transcribed and deductively analyzed in NVivo. Qualitative description was employed to describe study findings as barriers and facilitators to health equity integration.

**Results:**

Eleven individual interviews and one joint interview (*n* = 5 participants) were conducted, a total of 16 participants. Half (*n* = 8) of the participants were High-level Leaders (i.e., manager or higher) within the health system. We found that existing strategies within the health system were inadequate to address inequities, and variation in the use of indicators of health equity was indicative of a lack of health equity integration. Applying the CFIR allowed us to identify barriers to and facilitators of health equity integration, with the power of legislation to implement a Health Equity Framework, alongside the value of partnerships and engagement both being seen as key facilitators to support health equity integration. Barriers to health equity integration included inadequate resources devoted to health equity work, a lack of diversity among senior system leaders and concerns that existing efforts to integrate health equity were siloed.

**Conclusion:**

Our findings suggest that health equity integration needs to be prioritized within the health service and delivery system within Nova Scotia and identifies possible strategies for implementation. Appropriate measures, resources and partnerships need to be put in place to support health equity integration following the introduction of the Health Equity Framework, which was viewed as a key driver for action. Greater diversity within health system leadership was also identified as an important strategy to support integration. Our findings have implications for other jurisdictions seeking to advance health equity across health service and delivery systems.

**Supplementary Information:**

The online version contains supplementary material available at 10.1186/s12939-024-02256-7.

## Background

### The pursuit of health equity

The pursuit of health equity has been a priority of global public health and health systems for decades [1]. Health equity is defined as the ability for all individuals to attain their full potential for health and well-being [2]. Health equity therefore occurs when all people, irrespective of their race, ethnicity, gender identity, ability, socioeconomic status, or other demographic or geographic factors, have fair and just opportunities to attain or reach their maximum health potential or highest level of health [3, 4]. Differences in health status or outcomes among population groups stem from intersecting social, economic, or political processes that influence access to healthcare, making the need to promote and achieve health equity an important action for health service and delivery systems globally [4]. Indeed, the US Institute for Healthcare Improvement (IHI) incorporated health equity as an explicit goal to improve health system performance, which became known as the quintuple aim [5].

### Integrating health equity

If *achieving* health equity is the desired outcome of health care and delivery systems, what does the *integration* of health equity look like in practice? By health equity integration, we mean how actions, strategies or policies to achieve health equity become embedded throughout health service and delivery systems in ways that are coherent and identifiable across all levels of the system. According to the World Health Organization, health systems are all organizations, people, and actions whose primary intent is to promote, restore, or maintain health [6]. Health service and delivery systems, as a component of the health system, represent the “organization of people, institutions, and resources to deliver health care services to meet the health needs of a target population, whether a single-provider practice or a large health care system” [7]. Integrating health equity into health service and delivery systems therefore means developing and implementing strategies that are designed to reduce health inequities across all elements of the system [8]. For example, this could be achieved by ensuring that health equity is considered in hiring practices, so that the people who work in a health service and delivery system look like the population being served, and that policies are in place to ensure diversity in the workforce [9]. Other examples of health equity integration include collecting data on who gets seen in different components of a health service and delivery system and who is missing, or whether policies that guide routine clinical practice are equitable in their development and application [10]. Unfortunately, the ways that health equity is integrated are not well documented. We recently completed a scoping review that explored how health service and delivery systems in high income countries define and operationalize health equity, and identified the implementation strategies and indicators being used to integrate and measure health equity [11]. We found that strategies to advance health equity work were often siloed within health service and delivery systems and not integrated system-wide. We also found that the health equity definitions and frameworks that were used in the literature were varied, while indicators for health equity were often inconsistently measured [11]. We concluded that more evidence is needed on the integration of health equity across the entire health service and delivery system, as most work to date to advance health equity has focused on public health settings, health care providers, or health care delivery within a specific component of the health system [11].

### Understanding health equity integration in health services and delivery systems through implementation science

The ways that health equity is integrated into the different components of the health service and delivery system can be better understood through the lens of Implementation Science (IS). IS has been defined as the study of methods to promote the adoption and integration of evidence-based health interventions (e.g., tools, programs, and policies) into practice [12]. The Consolidated Framework for Implementation Research (CFIR) is a commonly used IS framework that can guide the systematic assessment of multi-level implementation contexts to identify or explain barriers and facilitators that might influence intervention implementation and effectiveness [13]. The CFIR is comprised of 39 constructs that operate across five domains (Innovation, Outer Setting, Inner Setting, Individuals, and Process) [13]. The CFIR has been applied to a range of contexts within health systems, ranging from individual programs to improve service delivery, to interventions to change health care provider practice [14]. Adaptations to CFIR have been made since it was first introduced, [14] but to our knowledge the model has not yet been applied to study health equity integration across health service and delivery systems.

### Context to this study

In 2022, the Government of Nova Scotia, Canada released a strategic health plan that included a commitment to address the health inequities faced by Nova Scotians within publicly-funded institutions such as education, justice and health care [15]. A key outcome of the strategic health plan, called Action For Health, was the development and release of a Health Equity Framework (HEF) in July 2023 to “guide targeted approaches on health equity experiences for various equity-seeking populations” [15]. The HEF also included a commitment to improve data collection to advance health equity, in recognition that all people do not have equal opportunities to reach their full health potential [15, 16]. Given the identified gap in the literature around how health equity is integrated across health service and delivery systems, [11], and ahead of the release of the HEF as part of the Dismantling Racism and Hate Act (2022), [17], we conducted qualitative interviews with provincial and regional health system decision makers. We sought to (1) explore how participating decision makers were currently integrating health equity within their areas of work of the provincial health system, and (2) through the application of the CFIR, to identify and describe key barriers and facilitators to system-wide health equity integration.

## Methods

### Setting

This study took place in the Atlantic Canadian province of Nova Scotia, home to approximately 1 million people. Key aspects of the provincial health system have been described in detail elsewhere [18]. Briefly, the system consists of six health system partners. The day-to-day organization of health care and public health is delivered by two health authorities. Nova Scotia Health (NSH), which was formed in 2015, is the provincial health authority that was created after the dissolution of nine district health authorities which previously governed most health service delivery within the province. IWK Health, which serves women and children across the Maritime provinces is the second health authority in Nova Scotia. The Department of Health and Wellness (DHW) is a provincial government department that provides strategic direction to the operations of both NSH and IWK Health. The Department of Seniors and Long-Term Care, the Office of Addictions and Mental Health, and the Office of Healthcare Professionals Recruitment are the remaining health system partners.

### Participant recruitment

Purposive sampling was used to recruit health system staff and/or decision makers who identified as (a) working within any of Nova Scotia’s health system partners, and (b) working on health equity initiatives or holding a leadership position within any of the health system partners. Leadership positions were categorized as holding a manager position or higher. High-level leaders were positioned at the director level or higher within Nova Scotia’s health system. Participants were identified using existing networks of the research team and/or public records and were invited to participate via an email sent by the principal investigators (SFLK or SMS). Interested individuals then contacted the research team directly to receive the study information and to schedule an interview. This research was conducted in accordance with the Canadian Tri-Council Policy Statement for Ethical Conduct for Research Involving Humans, which is in alignment with the Declaration of Helsinki. Ethics approval for this study was obtained from Nova Scotia Health Ethics Research Board (REB#1027909). All individuals who contacted the research team were deemed eligible to participate. Eligible participants were provided with a study information and consent form and either returned the signed form or provided oral assent following a study recap at the beginning of their interview.

### Data collection procedures

Eleven one-on-one interviews were conducted from February to June 2023. In addition, five participants working within the same department opted to participate in a joint interview that was held in May 2023. All interviews were facilitated by the research team (JY and SFLK) and recorded using Microsoft Teams videoconferencing software with participant consent. A semi-structured, open-ended interview guide was used to facilitate discussion, drawing upon evidence derived from the literature identified in our recent scoping review [11, 19]. The interview questions were designed to stimulate discussion about health equity integration by focusing on three key thematic areas: (i) their perceptions of health equity within their roles or areas of work, (ii) health equity strategies or indicators being used, as a means of understanding the extent of health equity integration (i.e., what indicators were being collected to measure whether health equity was being achieved within their area of work), and (iii) whole system approaches to health equity integration (see Additional File 1). Participants’ length of employment within the health system and the current area of work (portfolio) were also captured during the interviews. Interviews lasted approximately 45–60 min.

### Data analysis

Data analysis was guided by the thematic areas outlined above and by the domains of the CFIR [13]. Within the context of the CFIR, we considered health equity as the Innovation of interest, because it represented what was being implemented to achieve the outcome of health equity. The key components of the CFIR as they related to this study are depicted in Table 1.

**Table 1:** Contextual application of the components of the Consolidated Framework for Implementation Research [13]

CFIR Domain	Definition	Application to current study
Innovation	The “thing” being implemented	Health equity integration, defined as how health equity actions, strategies or policies are actioned or embedded throughout health service and delivery systems in ways that are coherent and identifiable across all levels of the system
Outer Setting	The setting in which the Inner Setting exists	Nova Scotia, Canada
Inner Setting	The setting in which the Innovation is implemented	Nova Scotia’s Health System, including the Department of Health and Wellness, Department of Seniors and Long-term Care, Nova Scotia Health, IWK Health, Office of Mental Health and Addictions, Office of Healthcare Professionals Recruitment
Individuals	The roles and characteristics of individuals	Individuals hired within the Inner Setting and individuals who receive care within the Inner Setting
Implementation Process	The activities and strategies used to implement the Innovation	Activities and strategies used to support health equity integration across Nova Scotia’s health system

Audio recordings of individual interviews and the joint interview session were transcribed using MS Teams, reviewed for accuracy by two of the authors (JY and SFLK), de-identified and then uploaded into NVivo (Version 12). Transcripts were coded by JY according to the principles of deductive content analysis [20] and were then mapped against the domains outlined in the CFIR [13]. To ensure consensus was achieved in terms of the coding process, meetings were held with two members of the research team familiar with the CFIR (ME and SMS) to review the codes created in NVivo against its domains.

A total of 16 individuals participated in this study (*n* = 11 in individual interviews, *n* = 5 in joint interview). Participants included high-level leaders (*n* = 8), mid-level leaders (*n* = 3), implementation leads (*n* = 2), implementation facilitators (*n* = 2), and other implementation support personnel (*n* = 1). On average, participants had worked within the health system for 12 years (range: 2–28 years) and within their specific area of work for five years (range: 1–17 years).

## Results

**Fig. 1:**
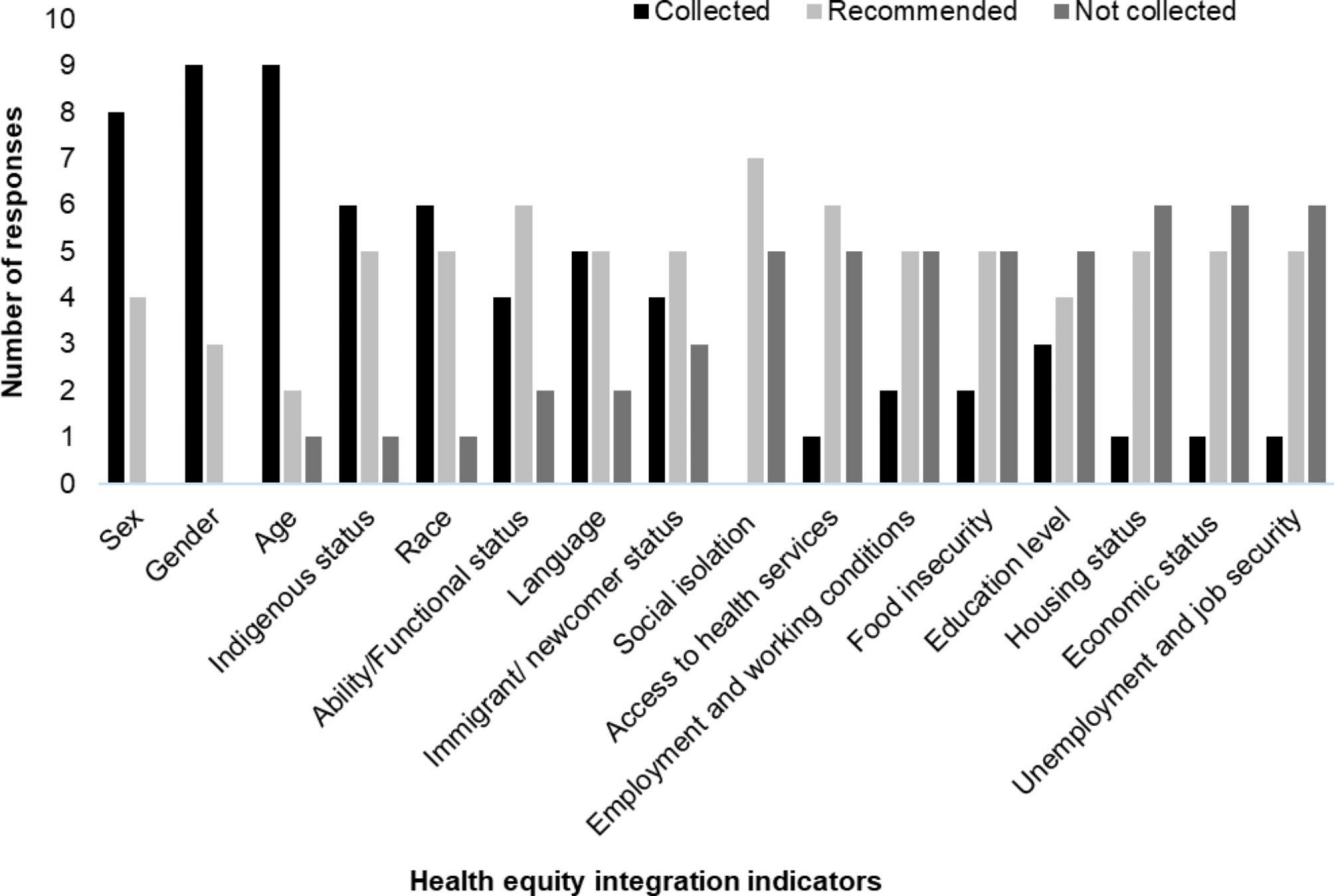
Participant reports of health equity indicators that are currently collected, recommended, or not collected in the health system, as a measure of health equity integration

Our results are structured according to the key thematic areas outlined above, starting with the health equity strategies or indicators that participants described as being used or needed as this provided concrete examples of whether or how health equity was being integrated (thematic area ii). Identified health equity indicators were categorized as “Collected” (currently being collected), “Recommended” (currently not being collected but participants felt should be collected), or “Not collected” (not being collected and/or not perceived to be viable by participants) (see Fig. 1). The most reported “collected” indicators were gender and age (each mentioned by nine participants), sex (mentioned by eight participants), Indigenous status and race (each mentioned by five participants) and language (mentioned by four participants). Indicators that were “recommended” most commonly were social isolation (mentioned by seven participants); ability/functional status and access to health services (mentioned by 6 participants respectively); and Indigenous status, race, housing status, immigrant/newcomer status, economic status, employment and working conditions, unemployment and job security, language, and food insecurity (each mentioned by five participants). Indicators “not collected” were housing status, economic status, and unemployment and job insecurity (each mentioned by six participants). Figure 1 illustrates that there was variation in the indicators that were collected across participant areas of work, suggesting a lack of consistency across the system around relevant or available indicators, which in turn suggests a lack of health equity integration.

Next, we explored perceptions of health equity (thematic area i) and whole systems approaches to health equity integration (thematic area iii) by mapping the relevant interview data onto the domains of the CFIR. This allowed us to identify barriers and facilitators to health equity integration across the Nova Scotia health system areas, as represented by our participants (Table 2). Below, we describe our results according to the domains of the CFIR.

**Table 2:** Summary of key barriers and facilitators mapped to CFIR domains

CFIR Domains	Key Findings	Illustrative Quotes
Barriers		
Innovation Complexity Design	The complexity of the Innovation was exemplified by how it is understood across the system and its time intensive nature. The design of the Innovation was not ready for implementation. This was evidenced in how participants discussed a lack of established, formal processes for implementation.	*“You know*,* some people feel like*,* we’ll put this in and in two or three years*,* we’ll start seeing a change*,* we will*,* but an overall change in something that’s been built for 50–60 to 100 years doesn’t change overnight.”* (Participant 10) *“The way we define it in terms of an ultimate outcome*,* as in long*,* long*,* long term is to*,* I guess*,* eliminate or reduce barriers*,* or eliminate or reduce disparities in health outcomes specifically. So that’s why we’re doing this course* [of action], *to get there requires probably a generation’s worth of work in terms of system change*,* which is what this framework is intended to start in a system-level way.”* (Participant 3) “*We have limited but some designated positions within the organization*,* but I’ll be frank and say*,* I don’t know that if you were to*,* I mean*,* honestly*,* like you’re asking me as an individual*,* I don’t know that there’s a formal process or procedure or framework to work within that would say*,* this is how I make sure that equity is included or considered in my decision-making in my hiring processes.”* (Participant 4) *“Pockets of that work do exist*,* of course*,* within each of the organizations that I mentioned*, [six health system partners] *but up until probably starting in September* [2022] *or so there weren’t really any mechanisms*,* within the exception of a few specific things*,* there weren’t many mechanisms for collaboration or conversation*,* communication across the health system. So*,* this is intended to*,* to bring all that together.”* (Participant 3)
Inner Setting Mission Alignment Available Resources	Participant’s discussions of how and whether the Innovation aligned with the Inner Setting mission and values was inconsistent. Leaders discussed implementation within their areas of work as occurring separately to the broader Inner Setting objectives. The perception of availability of resources was such that resources were limited and data quality and access, financing, and staff availability and positioning acted as a barrier to Innovation implementation.	*“Yeah*,* I would say right now*,* it’s because it’s not a priority*,* other than the* [HEF] *work.”* (Participant 1) *“It is 110%*,* so 110%*,* it is. We knew when we were asked to build this program*,* that*,* we had to do it in a way that would be meaningful to Nova Scotians good use of resources and build that trust. So from the very beginning*,* we paid attention to things like*,* like health literacy*,* reducing barriers.”* (Participant 9) *“So we strive to espouse principles associated with [Equity, Diversity and Inclusion, or EDI]*,* we happen to have a fairly varied team. So*,* our team might be more representative of various communities than other teams in the* [organization].*”* (Participant 4) *“Now for me*,* specifically*,* within my portfolio*,* I’ve taken it upon myself to do things that are a little bit*,* kind of*,* out of the box*,* you know*,* things that folks haven’t decided to do within their own ranks but for me*,* I*,* actually knowing how diverse*,* we are moving in that direction*,* felt the need about two and a half to three years ago*,* to start a respectful workplace program.”* (Participant 7) *“Yes. Resources*,* people resources*,* human resources are an issue because*,* you know*,* you don’t have enough people to do the work.”* (Participant 10) *“They’re going to have to decide*,* you know*,* can we reorient an existing system to do that? Can we? Or do we need to kind of tear something down and build up an entirely new structure in order to inform that work. And of course*,* if they decide either of those things*,* there’s implications for resources*,* both human and financial. And as you know*,* the health system is already very*,* very stretched*,* even on the administrative side. And so there’s some pretty significant implications for those*,* the resourcing of that.”* (Participant 3) *“Yeah*,* so we try to map it against a variety of different data sets*,* again to basically*,* to proxy the fact that we don’t like within health care…we don’t survey extensively around individuals. You know*,* we provide care*,* 99% of the information my team uses is for secondary use.”* (Participant 4)
Individual Leaders	Participants perceived Leaders as a barrier to Innovation implementation due to a lack of lived experience and a lack of understanding of health equity and related concepts.	*“I think it’s very difficult for folks without lived experience to understand how to integrate it. And so*,* I think that’s what keeps it from being incorporated into it*,* as it should be – into our organization*,* into my portfolio – is because I don’t think the people who are kind of leading the ship*,* understand what that looks like. So*,* they can’t solve a problem.”* (Participant 1) *“It’s very much like*,* oh*,* well*,* we have to work within* [organization], *or we have to work within* [organizational district], *or*,* oh*,* how does that relate to health? So I find like*,* I’m constantly having to make a case for things and I’m*,* you know*,* I’m going okay*,* like you’ve said*,* you want to do* [EDI] *work*,* and you said you*,* you know*,* want to think about the social determinants of health but when I bring things forward*,* it’s like*,* um*,* how does that relate to health?”*(Participant 2) *“Now we have no board*,* we have a very different executive*,* we have many new leaders in new positions who don’t have historical knowledge or awareness of the work that was done pre-pandemic.”* (Joint Interview participant)
Facilitators		
Outer Setting Partnerships and Connections Policies and Laws	The health system actively sought to foster and build partnerships with entities outside of the Inner Setting to improve design and implementation of the Innovation. The Dismantling Racism and Hate Act was a driver of the HEF and system-wide efforts to integrate health equity principles.	*“We have working groups*,* so we have the primary reference working group*,* which has been a fabulous engagement tool to connect with organizations outside of the Health Authority*,* or out of the DHW. So the primary reference group working group is representatives of equity groups*,* across Nova Scotia.”* (Participant 10) *“We listen deeply. So if we’re in a community*,* and it doesn’t go as we planned*,* how do we learn from that and do it better? We work with right now*,* you know*,* 250 community groups who are delivering rapid tests on behalf of governments to libraries*,* MLA offices*,* feed Nova Scotia and so we are constantly in relationship through*,* you know*,* regular check-ins*,* giving them what they need*,* reducing the barriers and making it easy for them to support that work.”* (Participant 9) *“We used to do those regularly* [use population-based health measures to understand health inequities] *when we were* [previous regional structure], *but now that we’re Nova Scotia Health*,* that kind of understanding of the health inequities*,* it’s kind of fallen by the wayside*,* and then COVID*,* everything got chucked out. So*,* it’s starting to get back on track. Now*,* perhaps the Health Equity Framework will help bring some of this back into the forefront.”* (Participant 1) *“So I think I’m very*,* very optimistic*,* where things are heading with the* [HEF] *right now becomes a barometer for us to take away and use to*,* you know*,* a lens to a tool to use across the system.”* (Participant 11) *“Basically*,* the Health Equity Framework*,* all health systems*,* service delivery partners*,* including addictions and mental health*,* healthcare*,* professional recruitment*,* seniors in long term care*,* Nova Scotia Health and IWK will be essentially beholden to the Framework*,* as in they are required to deliver on that and they are required to be accountable for that. So once the Framework is released*,* that’s going to be the status of things so it will guide all equity related action across the system.”* (Participant 3)
Engagement – across multiple domains	Engagement through developing external Partnerships and Connections indirectly inform the Innovation through influencing design components. Engagement was used as a process for developing Relational Connections across the Inner Setting. As an Implementation Process, engagement with participants was used to inform health equity integration.	*“So we have to find a way to engage very well and one of the ways of doing that is talking to Nova Scotians where they are so we have to know the makeup of our communities. Like I said*,* we’re growing a lot around the immigration side of things*,* so just last week or so we went to the* [Religious association in Nova Scotia], *have a conversation at the* [Religious place of worship], *talk to the* [Religious leaders] *learning about some issues around social determinants of health.”* (Participant 11) *“So*,* some of the work around equity lands at some of the EDIRA [Equity*,* Diversity*,* Inclusion*,* Reconciliation*,* Accessibility] tables or the disk tables*,* diversity and inclusion tables of which every zone has one and it’s a*,* they’re made up of folks from across the health system and health system departments”.* (Joint Interview Participant) “*And we’re kind of behind the scene*,* learning from them and kind of say*,* hey*,* when you ask this*,* can we ask kind of reach out to your*,* to your network*,* to your communities I had*,* that’s how we built kind of our network with the community and community partners.”* (Participant 6)

### Barriers to health equity integration

#### Innovation complexity

Participants highlighted the complexity of the Innovation as a barrier to successful implementation, because of the size and scope of what was needed to integrate health equity. This was summarized by Participant 10 who stated “*some people feel like*,* we’ll put this in and in two or three years*,* we’ll start seeing a change*,* we will*,* but an overall change in something that’s been built for 50–60 to 100 years doesn’t change overnight”*. This participant conveyed a broadly held misperception within the system that change will occur directly following implementation of the HEF, noting that “*It’s really also challenging to tell people*,* if you do this*,* it will make an impact*,* but I can’t show you right away”.* Participant 3 described achieving the outcome of health equity as a “*generation’s worth of work*,” noting that the HEF “*is intended to start* [this work] *in a system-level way”*. Thus, the complexity lies in health equity integration requiring ‘faith’ that it will happen rather than demonstratable outcomes that may not be seen within a short timeframe.

### Innovation design

Participants discussed that Innovation implementation was in progress, but lacked an established, formal process for implementation. This was discussed by Participant 4, who noted that the design of the Innovation was “*aspirational but not yet operational*”, with uncertainty around hiring of designated positions being given as an example of this: “*I don’t know that there’s a formal process or procedure or framework to work within that would say*,* this is how I make sure that equity is included or considered in my decision-making in my hiring processes”*. Participant 4 further questioned the design by suggesting that a lack of a “*clear direction*,* or objective within the realm of equity”* existed across the system. For Participant 3, the design was felt to be still in progress, that an integrated system “*does not exist”*, and that “*up until probably starting in September* [2022] *… there weren’t many mechanisms for collaboration or conversation*,* communication across the health system. So*,* this is intended to*,* to bring all that together”*. Thus, Innovation design posed a barrier due to the evolving nature of the work (i.e., the development of the HEF) that had not yet shifted health equity integration from aspirational to operational. The aspirational nature of the Innovation Design was further exemplified by a lack of a formal process for implementing Innovation components, such as hiring for designated positions.

### Inner setting: mission alignment

Participants offered inconsistent accounts around the alignment of the Innovation with the mission of Inner Setting partners. For example, Participant 1 stated the Innovation is *“not a priority*,* other than the [HEF development] work”*. Meanwhile, Participant 9 felt that *“it is 110%*,* so 110%*,* it is”*. Participants who were either Mid or High-level Leaders also noted that Innovation implementation within their areas of work occurred separately to the mission of the Inner Setting. For example, Participant 4 stated that within their area of work *“we try to espouse principles* [of health equity]*”*, citing the diversity in their team as *“more representative of various communities than other teams”* in the system. For Participant 7, it was through their own personal motivation and initiative that action toward Innovation implementation took place within their area of work:

*I’ve taken it upon myself to do things that are a little bit*,* kind of*,* out of the box*,* you know*,* things that folks haven’t decided to do within their own ranks but for me*,* I*,* actually knowing how diverse we are*,* moving in that direction*,* felt the need about two and a half to three years ago*,* to start a respectful workplace program* (Participant 7).

Participants who were not leaders also reflected on a disconnect between the Innovation and goals and objectives of the Inner Setting. Participant 5 noted that the failure to align the Innovation with the mission of the health system resulted in health equity being considered an “add-on” rather than a necessary component of operations, saying: “*programs*,* strategies*,* and directions don’t explicitly identify how they are addressing equity*,* diversity*,* and inclusion in their day-to-day work”*. Participant 5 thus perceived the lack of mission alignment as a causal mechanism for the lack of explicit consideration of the Innovation within implementation processes and noted the importance of equity being an organizational goal.

For Participant 9, who was tasked with developing and implementing a novel program, there was an opportunity to ensure explicit consideration of the Innovation, *“we knew when we were asked to build this program that we had to do it in a way that would be meaningful to Nova Scotians and a good use of resources and build that trust”*. Participant 9 further identified how their team gave explicit consideration in program development to “*health literacy*”, “*reducing barriers*”, and “*providing information in a variety of ways*”. Overall, these reflections suggest there were contradictory perspectives on the Innovation’s alignment with the mission of the Inner Setting that could impact successful integration of health equity. Fragmented mission alignment may also be indicative of silos across Nova Scotia’s health system.

### Inner setting: available resources

The CFIR defines the available resources construct as “the degree to which resources are available to implement and deliver the Innovation” (p.6), and provides funding and space as the subconstructs [13]. For our purposes, resources were considered any input required to improve the success of Innovation implementation and included staff, funding, training and data. The availability of resources was frequently discussed as an existing barrier to Innovation implementation. Participant 10 noted that *“resources*,* people resources*,* human resources are an issue because*,* you know*,* you don’t have enough people to do the work”*. Participant 3 expressed a similar sentiment, noting that there was strain across the Inner Setting, such that with any Innovation work, particularly surrounding the HEF, there would be significant implications for resources:

*They’re going to have to decide*,* you know*,* can we reorient an existing system to do that? Can we? Or do we need to kind of tear something down and build up an entirely new structure in order to inform that work. And of course*,* if they decide either of those things*,* there’s implications for resources*,* both human and financial. And as you know*,* the health system is already very*,* very stretched*,* even on the administrative side. And so there’s some pretty significant implications for those*,* the resourcing of that*.

Participants who were not leaders but who supported the implementation of the Innovation (e.g., implementation leads and implementation facilitators) noted their siloed positioning and lack of financial resources as barriers, as illustrated by Participant 5: *“Right now*,* as you can tell*,* we don’t have a diversity*,* equity*,* and inclusion structure within the organization*,* it’s one person led work”*. They went on to say that: *“Thankfully*,* we have an* [implementation lead position], *they started about 3 years ago…but we are at different*,* we are placed at different*,* what is it? Structures within the organization. We are not* [under] *the same umbrella or roof”.* This further illustrates the siloed nature of the work and a need for greater mission alignment.

Data access was also noted as a barrier to Innovation implementation. Participant 1 described handling data related to health equity as akin to “*analytic gymnastics*” to formulate proxies for measures, something that Participant 4 also referenced, saying: *“Yeah*,* so we try to map it against a variety of different data sets*,* again to basically*,* to proxy the fact that we don’t like within health care…we don’t survey extensively around individuals. You know*,* we provide care*”. Participant 4 went on to quantify the extent that data access posed a barrier, stating “*99% of the information my team uses is for secondary use”.* Within the Inner Setting, therefore, there is a need for greater mission alignment and for this to be accompanied by adequate resources – human and in terms of data – to advance health equity integration.

### Individual domain: leaders

Barriers within the individual domain were primarily concerned with leadership across the Inner Setting. Specifically, the perceived capability of leadership to implement the Innovation was frequently questioned, due to a perceived lack of diversity among system leaders. This was summarized by Participant 1 as: *“I think it’s very difficult for folks without lived experience to understand how to integrate it. And so*,* I think that’s what keeps it from being incorporated into it*,* as it should be – into our organization*,* into my portfolio”*. Participant 1 provided a rationale for the lack of integration stating that it “*is because I don’t think the people who are kind of leading the ship*,* understand what that looks like. So*,* they can’t solve a problem”*. This participant further noted how most of the senior leadership were “*cis-straight-white”* and that health equity “*is not on their radar. It’s seen as an aside”.* Participant 2 also noted a perceived lack of understanding among leadership and a tension between the need to deliver healthcare and a broader understanding of the social determinants of health, which were viewed as lying outside of, and therefore unrelated to health. Participant 4 further noted the current understanding of the Innovation by leadership was not at a level needed to support implementation, suggesting *“the largest barrier is a shift in mindset”* of high-level leadership to recognize that *“it is the right thing to do”.*

Leadership was also discussed in the context of external shocks that had impacted the health system, specifically the COVID-19 pandemic and a change in provincial government in 2021 which led to some restructuring within the health system leadership. One of the joint interview participants noted that *“Now we have no board*,* we have a very different executive*,* we have many new leaders in new positions who don’t have historical knowledge or awareness of the work that was done pre-pandemic”.* Evident here is the perception of a two-fold barrier, one specific to the change in the role of leadership and one specific to leadership capabilities due to a gap in institutional memory.

Participant 5 commented specifically on the role/position of leadership across the provincial health system rather than the specific capabilities of high-level leadership, suggesting a need to change performance evaluation metrics of leadership: *“I think one thing is leadership accountability*,* added accountability*,* and who holds them*,* to the… to the task*,* and how are they held to that task? So*,* it needs to be clear and within their job descriptions”*. This participant suggested that without structured accountability frameworks and organizational directives, Innovation implementation will not succeed.

### Facilitators of health equity integration

Within the Outer Setting domain, Partnerships and Connections and Policies and Laws were identified as important facilitators of the Innovation. Engagement was discussed by participants as a facilitator to breaking down silos across the system and a pivotal driver of health equity integration.

### Outer setting: partnerships and connections

Participants indicated the importance of Partnerships and Connections to the Inner Setting. The Partnerships and Connections construct is “the degree to which the Inner Setting is networked with external entities, including referral networks, academic affiliations, and professional networks” (p.5) [13]. Participant 10 noted the establishment of a “*primary reference working group*,* which has been a fabulous engagement tool to connect with organizations outside* [of the Inner setting]*”*. This working group was described by Participant 10 as *“representatives of equity groups across Nova Scotia”* that supported a specific equity initiative, including the development of a data governance structure and a process of oversight. This structure therefore represented a powerful and sustainable connection between the Inner Setting and external entities, thereby allowing diverse perspectives to influence the Innovation.

Participant 11 also described how the Inner Setting had established Partnerships and Connections with a variety of external groups, including health professional organizations. These were illustrative of a tiered network of external partners (i.e., community groups who then distribute information or resources to another external partner). Overall, discussions indicated the significant extent to which the Inner Setting was networked with external entities and the foundational role of Partnerships and Connections to the Innovation.

### Outer setting: policies and laws

Nova Scotia’s HEF was released after these interviews were conducted. Through our interviews with health system partners, we identified that the HEF’s impending release, as part of the Dismantling Racism and Hate Act (2022), was considered a key driver in facilitating Innovation implementation within the health service and delivery system. Participant 1 reflected on how with a shift in organizational structure, population-based health measures had *“kind of fallen by the wayside*,* and then COVID*,* everything got chucked out”*. Despite this, Participant 1 had room for optimism stating that “*perhaps the [HEF] will help bring some of this back into the forefront”.* Other participants noted the importance of legislation to advance the HEF and increase accountability, thereby reducing and eliminating factors that had previously hindered achievement of health equity within the health service and delivery system, with Participant 11 sharing optimism that the HEF *“right now becomes a barometer for us to take away and use to*,* you know*,* a lens to a tool to use across the system”.* Participant 3 further noted that *all health* system *partners* would *be “beholden”* to the HEF “s*o once the Framework is released*,* that’s going to be the status of things*,* so it will guide all equity related action across the system”*. Thus, by outlining actions and key focus areas to address the current inequities within the health service and delivery system, the HEF was seen as critical to advancing health equity integration and achieving the outcome of health equity.

*Engagement – An Innovation Facilitator Across Multiple Domains Towards a Whole Systems Approach to Health Equity Integration*.

When asked about system wide considerations for health equity integration, Engagement was frequently discussed as an Innovation facilitator across multiple domains of the CFIR. The foundational role of engagement in health equity was highlighted by Participant 3 who stated that *“And I mentioned that because of course*,* you can’t really do equity work without engagement”*. Within the Outer Setting, engagement was evident in the extent that Partnerships and Connections were a product of efforts to engage external entities into the Inner Setting. One example of this is the reference group with diverse representation that was previously discussed.

Given the role of external entities in development and implementation of aspects of the Innovation, engagement through Partnerships and Connections indirectly influenced the Innovation domain through informing design. Participant 9 described engagement as foundational to equity work with external entities by stating “*it’s all about relationships*” and that creating relationships can be achieved through “*providing regular information*,* keeping in touch regularly with other services*,* making sure that they have accurate*,* current information to be able to be able to help navigate and support us and do our work together*”.

Across the Inner Setting, participants discussed engaging through relational connections and work related to the HEF. For example, *the* joint interview Participants indicated engagement across the Inner Setting throughout their discussions, noting how: *“some of the work around equity lands at some of the EDIRA [Equity*,* Diversity*,* Inclusion*,* Reconciliation*,* Accessibility] tables or the diversity and inclusion tables…and it’s a*,* they’re made up of folks from across the health system and health system departments”.*

Engagement was also discussed as a mechanism for supporting implementation processes by participants. Participant 10 provided an example of engaging with recipients to bring *“lived experience to the table”* after noting it was *“the biggest thing we need to do”* when discussing how mental health facilities shared similarities *to* a *“jail”*, where *the ”doors have two or three locks”*, *“other rooms don’t have windows but all the doors have windows”*, and there *“are some bars”* and how this had implications for populations that have been marginalized. Specifically, Participant 10 tied the infrastructure to the lived experience by stating that *“you feel like you’re going back to jail and if that is the place that you’ve had your issues with mental health*,* that’s not going to be very welcoming”.* Evidently then, lived experience was viewed as crucial in developing physical environments that reflect equitable decision-making.

Participant 6 also noted Inner Setting engagement was used to reach Outer Setting partners who may be more familiar with certain Inner Setting partners by stating “W*e’re kind of behind the scenes*,* learning from them and kind of say*,* hey*,* when you ask this*,* can we kind of reach out to your network*,* to your communities… that’s how we built our network with the community”*. Inner Setting engagement in this sense therefore acted as a mechanism for reducing Outer Setting engagement fatigue.

While engagement was widely discussed as an Innovation facilitator across multiple domains, some participants identified challenges with engagement. For example, Participant 5 noted that health equity in programs and services within the Inner and Outer Settings were “*siloed*” stating that now there are “*no clear pathways for these collaborations”*. Participant 2 further explicated this by stating “*it’s really about making sure that there’s collaboration*,* and I feel like that might be the piece that’s missing for me”*. Despite this challenge, overall discussions indicated the importance of engagement to facilitate the Innovation across all domains of the CFIR.

### Understanding of health equity integration

Participants expressed varied degrees of understanding around how to integrate health equity across the health system in Nova Scotia. This variance in understanding may reflect the variance in health equity integration into the roles that participants occupied. For example, Participant 9 described health equity as integrated into their role; whereas Participant 1 noted that it was not integrated into theirs (see Inner Setting: mission alignment). Consequently, Participant 9 expressed more positive comments toward health equity integration across the system compared to Participant 1. Conversely, the understanding of participants may reflect their personal experiences with marginalization. For example, Participant 2, who self-identified with *“a community that is marginalized”*, expressed that, due to this, *“there’s a little more self-situational awareness and I understand the sensitivity*” and as a result noted that they “*go above and beyond*” despite health equity not being explicit in their area of work.

While some participants were critical of the system’s attempts to integrate health equity, they also shared their personal commitment. Participants 2, 4, and 7 all noted how they strived to integrate health equity through their role but had questions about the way that the system operated to ensure Innovation implementation. For example, Participant 4 discussed the “*lack of an overall objective*” (see Inner Setting: mission alignment) while also discussing hiring efforts specific to their department to improve diversity. Participant 7 noted efforts towards “*hiring a diverse group*” as something that was done independently of broader system drivers.

Participants identified health equity integration as being influenced by existing power differentials across the health system. This was reflected in participant critiques of the lack of diversity among leadership. For example, Participant 4 discussed the need for *“a shift in mindset”* among leadership to recognize the moral importance of the Innovation’s implementation (see Individual domain: leaders). Approximately half of the participants perceived that the understanding among leadership of the Innovation, or the ability of leadership to implement the Innovation, was a barrier, whether because of lack of lived experience, strict adherence to the organizational structure and processes, or an inadequate knowledge of the Innovation.

## Discussion

### Summary of findings

This qualitative analysis of health equity integration within the health service and delivery system of Nova Scotia provides important insight into the conditions in place prior to the implementation of the HEF. As a marker of health equity integration, we described the range of indicators of health equity that were collected, recommended or not collected, that were identified by our participants. The variability in indicators reflects a lack of integration within the provincial health system prior to the release of the HEF. If health equity was being integrated effectively, we would expect to see an agreed upon set of indicators being used across all areas of work that were represented by participants. This was not the case, although the recently released HEF clearly provides a mechanism for this integration to happen, which was described by our participants. Through applying the CFIR domains, we identified several barriers and facilitators in the way that health equity was being integrated into the provincial health service and delivery system that have implications for policy, practice, and research. In addition, we highlighted current understandings of health equity across the health service and delivery system. These suggest that, overall, a unified understanding of the Innovation was found to be lacking across health system decision makers. We also identified the Outer Setting domain, with specific emphasis on Partnerships and Connections and Policies and Laws, as a key opportunity to support and sustain the Innovation. Below, we discuss the key barriers and enablers on health equity integration within the context of the broader literature.

### The power of legislation to drive health equity integration

We found that existing strategies for Innovation implementation were inadequate across the health system, but that the power of legislation was identified to be a primary driver of health equity integration. The barriers identified suggested a lack of unified understanding of the Innovation across the system and a misalignment of the goals of the health system with the goals of health equity integration. Participants noted how, at the time of this study, Nova Scotia’s health system lacked an accountability driver for health equity work that could impose a mandate on all system partners to contribute the resources necessary to support health equity goals. Health equity specific frameworks are beneficial to build shared understanding of fundamental health equity concepts across all partners within the health system, [21] and in the Nova Scotia context, the mandate imposed by the HEF was seen to create shared momentum across the system, provide guidance on achieving actionable goals, and serve as a plumbline to measure progress towards planned goals [21]. The Dismantling Racism and Hate Act (2022), [17] a first of its kind legislation in Canada, provided the impetus to integrate health equity into the province’s health system and has policy implications for other jurisdictions. The emergence of the HEF may therefore provide the Nova Scotia health service and delivery system with the needed structure to address mission alignment and redirect resources. Nova Scotia’s current provincial strategic plan, called ‘Action for Health’, also acted as a catalyst for the development of the Health Equity Framework [16]. These initiatives provided the conditions upon which progress on health equity integration strategies within the health system can be realized. Other provinces may benefit from similar provincial level mandates for health equity integration within their health service and delivery systems. It is important to note, however, that the release of the HEF alone does not guarantee measurable change, but it can be viewed as a starting point or a foundation towards achieving an equitable health system. Further investments are necessary to enhance the professional competencies of all partners involved and widen organizational capacity to facilitate health equity integration [22].

### Partnerships and engagement are a priority

The identification of the importance of Partnerships and Connections, and Engagement as key drivers of Innovation implementation within the health system has important implications for practice. Partnerships provide the lived experience perspectives that interview participants described as critical to advance health equity integration provincially [23]. Partnerships are often formed with organizations that act as the voice of populations facing health inequities that then advocate for more equitable health systems [24]. Partnerships must therefore be valued as they provide a platform for local champions of health equity and function as mediators between communities and health systems [25]. Boothroyd et al. highlighted that partnerships are significant as they build relationships by engaging with communities, understand their issues and impose pressure on the system to address existing barriers [26]. Through their engagement in continual conversation with the health system to ensure support and services are appropriately delivered, Partnerships and Connections in the context of the CFIR indirectly hold systems accountable for Innovation implementation and help co-create implementation infrastructure for the Innovation. Engagement can be strengthened by exploring how often recipients across all communities have opportunities to voice their concerns, how well they use these opportunities, and any barriers associated with them [24]. This is significant as community organizations with developed partnerships, as noted above, can impose pressure upon the health system to integrate health equity strategies and processes [26]. However, based on the barriers identified in this and other studies, [24] it is evident that internal systems are not necessarily well set up to deal or cope with this pressure. Consequently, health equity integration needs to simultaneously prioritize engagement processes in Innovation design and address the barriers within the Inner Setting.

### Resources are required to support health equity integration

Our findings highlight the need for appropriate resources to be allocated to provide necessary training to build capacities and competencies within the system in a way that increases the relative priority of health equity, which has also been identified in research on addressing health inequities in primary health organizations from Australia [27] and Canada [28]. An analysis of the equity actions taken by public health nurses working within primary health networks noted the need for sufficient time and resources to meaningfully conduct health equity work, particularly in relation to implemented effective participatory planning processes [27]. Similarly, an evaluation of a provincial policy among health authorities in British Columbia emphasized the fundamental importance of prioritizing health equity within health systems to address health inequities, but also acknowledged the need for adequate support structures in place within the system to support effective prioritization [28]. Strategies involve building a culture of equity by embedding health equity as a priority across all areas of the health system. Prioritization of the Innovation within the health system could establish clearer lines of communication concerning the HEF’s intentions and emerging work, to ensure that the different working parts of the health system can collectively meet planned targets. The results-based accountability framework could be applied to examine progress of Innovation implementation alongside identifying factors that lie along the pathway to its execution [29]. In brief, this involves assessment of the effort and effects (of the Innovation) through quantifiable (what did we do? ) and qualitative (how well did we do it? ) measures [30]. This also reinforces the importance of data as a resource for decision-making to achieve health equity [31]. In our study, siloed data collection (i.e., health indicators) across different areas of work further suggested a disconnected approach within the system towards the Innovation. The organizational structure was also not adequately designed to support the transition to a more equitable system. Thus, appropriate resources were unavailable or insufficient to support Innovation implementation.

### Leaders lead the way

This study identified a perceived lack of lived experience of health equity issues and competence among those in high-level leadership positions. This is consistent with existing literature identifying leaders as important influencers to Innovation implementation [32, 33]. Diversity within leadership has long been identified as necessary to advance health equity actions [9, 34, 35]. Hiring individuals from diverse backgrounds may improve system-wide understanding of the inequities present in communities [36]. Diverse leadership helps to improve representation of marginalized communities in positions of power, bring insights into potential solutions to address inequities from a place of experience, and gives voice to knowledge of strategies that are beneficial or that have previously not worked [37]. Commitment within leadership, supplemented by the ability to interpret and act upon data relating to inequities, is essential to advance the Innovation within the health system [28]. Pauly et al. reported that leaders felt challenged when discussing issues around health equity because it is understood differently by others, and they often lacked support or guidance to integrate health equity within their work [38]. Leaders must be trained or upskilled to help build health equity competencies [38]. These supports will additionally help leaders facilitate prioritizing Innovation within their working departments. Future research could explore whether competency training in health equity can counter lack of lived experience and drive health equity integration. Furthermore, given the expressed importance of engagement as a tool for improving implementation across multiple domains, the role of different engagement tools (e.g., IAP2 Spectrum of Publication Participation) [39] to assess the level of engagement occurring across health systems is another avenue of inquiry.

### Strengths and limitations of the study

To our knowledge, this is the first study to explore health equity integration within the Nova Scotian health service and delivery system, and the barriers and facilitators associated with it. The use of qualitative methodology and semi-structured interviews fostered exploration of health equity integration in detail, given the scarcity of data in this area. Additional strengths include interviews with high-level leaders across different areas of work within the health system, which helped provide broader insights into existing health equity integration within the system. Leaders interviewed were individuals with decision-making power, who played key roles in the implementation of health equity across the health service and delivery system in the province at the time the study was conducted. The study was limited by the period during which data collection occurred, as it impeded participant recruitment, when the health system was still recovering from the impacts of COVID-19. Although all six health system partners were invited to participate, we did not have equal representation across the provincial health system. The CFIR was applied as an analytic framework post data collection. Future studies could consider applying the framework to the design and interview questions to strengthen study methodology. Furthermore, it is already established that context matters within IS interventions such as this one [13].

## Conclusion

Achieving health equity within the health service and delivery system is critical to improving health and reducing inequities faced by populations. In this study, we explored the integration of health equity within the Nova Scotian health service and delivery system through the perspectives of those in high-level leadership positions or who work directly to integrate health equity. We found that, while some evidence of health equity integration was observed, ongoing efforts to integrate health equity were siloed. Furthermore, health equity integration was not identified as a priority across the system at the time the study was conducted. We found that partnerships and engagement with communities facing inequities could exert pressure on the health system to address health inequities, but the internal structures and existing strategies were not designed in a way that could act upon these concerns. These findings have implications for researchers, practitioners and policy makers tasked with achieving health equity in health service and delivery systems by highlighting the barriers that limit progress towards this goal.

### Electronic supplementary material

Below is the link to the electronic supplementary material.

Supplementary Material 1

## Data Availability

The datasets generated and/or analysed during the current study are not publicly available to protect the identities of research participants but are available from the corresponding author on reasonable request in a deidentified format.

## Abbreviations

CFIR: Consolidated Framework for Implementation Research
DHW: Department of Health & Wellness
EDI: Equity, Diversity and Inclusion
EDIRA: Equity, Diversity, Inclusion, Reconciliation, Accessibility
HEF: Health Equity Framework
IS: Implementation Science
NSH: Nova Scotia Health
