# Supplementary material for: Exploring health equity integration among health service and delivery systems in Nova Scotia: perspectives of health system partners
Source: Int J Equity Health. 2024 Aug 26;23:171. doi: 10.1186/s12939-024-02256-7 (PMC11345956; doi:10.1186/s12939-024-02256-7)
Supplement: Supplementary file 1 — Supplementary Material 1 [file 12939_2024_2256_MOESM1_ESM.docx]

***Designing health service and delivery systems for impact in population health and health equity: What are the most meaningful indicators of success?***

**Interview Guide for Senior Leaders and Health Equity Focused Staff in NS Health**

**INTRODUCTION:**

The purpose of our interview with you, as a senior leader, is to explore the implementation of health equity strategies and indicators within Nova Scotia Health.

We define health equity as systemic, avoidable, and unfair differences in one or more characteristics of health[^[1]^](https://cac-word-edit.officeapps.live.com/we/wordeditorframe.aspx?new=1&ui=en-us&rs=en-US&wopisrc=https%3A%2F%2Fdalu.sharepoint.com%2Fteams%2FScRev-CC-SB%2F_vti_bin%2Fwopi.ashx%2Ffiles%2F59949c6db8af40a7adad961a60183564&wdenableroaming=1&mscc=1&hid=31fa1c21-37df-48d5-8698-724c2aaaf24f.0&uih=teams&uiembed=1&wdlcid=en-us&jsapi=1&jsapiver=v2&corrid=1bbcbce9-660b-4ed0-a63b-d0276286a87a&usid=1bbcbce9-660b-4ed0-a63b-d0276286a87a&newsession=1&sftc=1&uihit=UnifiedUiHostTeams&muv=v1&accloop=1&sdr=6&scnd=1&sat=1&rat=1&sams=1&mtf=1&sfp=1&halh=1&hch=1&hmh=1&hsh=1&hwfh=1&hsth=1&sih=1&unh=1&onw=1&dchat=1&sc=%7B%22pmo%22%3A%22https%3A%2F%2Fwww.office.com%22%2C%22pmshare%22%3Atrue%7D&ctp=LeastProtected&rct=Normal&wdorigin=TEAMS-ELECTRON.teamsSdk.openFilePreview&wdhostclicktime=1673378385474&wdredirectionreason=Unified_SingleFlush#_ftn1). These ultimately result in disparities in health that negatively affect the health outcomes of some populations. Those at risk for disparities resulting from health inequities include persons who are racialized, Indigenous, or marginalized because of other demographic, social, and/or economic factors e.g., sex, gender, income, ability, education, or access to health and social services

Our research team is interested in hearing your perceptions of health equity and its current status within Nova Scotia Health. For example, are policies and practices that support health equity currently in place? How has health equity been operationalized organizationally in addition to within your portfolio? If health equity policies and practices are not implemented, why do you think this is?

Your interview responses will be anonymous and only viewed by the research team. The results will be reported in an aggregated form and may be used to inform health equity work within Nova Scotia Health.

Are you still interested in participating and submitting a signed consent form? Are you willing to have your interview recorded? If not that’s fine as I will be taking notes throughout the interview.

If at any time you do not wish to continue participating, please feel free to end the interview.

**INTERVIEW QUESTIONS**

**Part 1: Perceptions of health equity as it relates to your role and services and programs within your portfolio**

1. Can you briefly describe your role? What department(s) does your portfolio include?

2. How long have you been working in your current portfolio?

- Less than one year
- 1-5 years
- 5-9 years
- More than 10 years

3. How long have you been with employed with Nova Scotia Health?

- Less than one year
- 1-5 years
- 5-9 years
- More than 10 years

5. How is health equity currently incorporated into the responsibilities of your position? (e.g., expand access, reporting, engagement and so on with populations at risk, etc.)

6. Is health equity an explicit consideration in the goals and objectives for the services and programs included in your portfolio?

6.1 If YES, briefly describe how?

6.2 If NO, based on your experience what prevents healthy equity from being incorporated within your portfolio?

**Part 2: Health equity strategies: Impact assessment and data collection**

7. What strategies are you using to understand which populations experience health inequities or are at risk for experiencing health inequities in the services and programs included in your portfolio? Interviewer can use list as a prompt and/or check all that are mentioned.

**PROMPT**: Some examples could include:

- - Health equity impact assessment
  - We engage with populations at risk e.g., ad hoc advisory meeting, survey, planning committee, etc.
  - Cross department work using whole system approach
  - There is a designated a health equity lead person / health equity team
  - Other please describe ______________________

7.1 For example, is health equity impact assessment an established practice within any/all of the services within your portfolio? If yes, please describe how health equity impact assessment is used? *(*Interviewer to check all that are mentioned or can use list as a prompt).

- Conducting annual operational review of a program and/or clinical service
- Conducting a quality review of a program and/or clinical service
- When making a revision or update to an existing program and or clinical service
- When planning or introducing a new program and/or clinical service
- As part of preparing for accreditation
- Other (please describe)

8. IF NO HEALTH EQUITY STRATEGIES ARE IN PLACE, what are the challenges of integrating health equity strategies within your portfolio?

9. Within your portfolio, are data on health equity characteristics collected which could identify populations that have experienced or are at risk of experiencing health inequities?

9.1 **If YES**, please describe how data are collected? How is this information used? (e.g., program planning, policy development, quality improvements)

Or, interviewer could ask about each of the following:

- - Standardized or organization-wide indicators as part of routine reporting on quality
    - Yes No
  - Standardized or organization-wide indicators as part of routine reporting on performance
    - Yes No
  - Standardized or organizational-generated indicators as part of accreditation report
    - Yes No
  - Standardized or organizational-generated indicators as part of a strategic plan
    - Yes No
  - Unique indicators collected by my service area/portfolio/program/department as part of reporting on quality
    - Yes No
  - Unique indicators collected by my service area/portfolio/program/department as part of reporting on performance
    - Yes No
  - Unique indicators collected by my service area/portfolio/program/department as part of reporting for a strategic plan
    - Yes No

9.2 IF NO DATA ARE COLLECTED, what are the obstacles that prevent collecting health equity data?

10. For each of the following indicators, please indicate whether or not you collect them and/or would like to collect them:

- - Sex
  - Gender
  - Age group
  - Education level
  - Indigenous status
  - Race
  - Ability/functional status
  - Housing status
  - Immigrant/newcomer
  - Economic status i.e., social assistance, disability or low income
  - Employment and working conditions
  - Unemployment and job security
  - Social isolation due to age, disability
  - Language
  - Food insecurity
- Access to health services
- Other (please identify)

**Part 3: Whole system approach to health equity**

11. If health equity work IS BEING DONE in your portfolio how does it connect to the work/efforts of other Nova Scotia Health departments?

12. If health equity IS NOT connected to the efforts of other departments what are the barriers which prevent this from happening?

13. If health equity work in your portfolio IS connected to organizations OUTSIDE of Nova Scotia Health please describe the strategies used to increase connections across the health system?

14. If health equity work in your portfolio is NOT CONNECTED to organizations outside of Nova Scotia Health, what are the obstacles that prevent creating connections across the health system?

15. What do health care organizations need to focus on (prioritize) to improve health equity integration? What is the role of Nova Scotia Health in this regard?

**CONCLUSION:**

That concludes the questions I have for you today. I appreciate your participation. If you have any more questions regarding the study, you can email me (JYusuf@dal.ca) or the co-principal investigators Dr. Sim ([meaghan.sim@nshealth.ca](mailto:meaghan.sim@nshealth.ca)) and Dr. Kirk ([sara.kirk@dal.ca](mailto:sara.kirk@dal.ca)).
